# Current Trends in Biomedical Hydrogels: From Traditional Crosslinking to Plasma-Assisted Synthesis

**DOI:** 10.3390/polym14132560

**Published:** 2022-06-23

**Authors:** Kathrina Lois M. Taaca, Eloise I. Prieto, Magdaleno R. Vasquez

**Affiliations:** 1 Department of Mining, Metallurgical and Materials Engineering, College of Engineering, University of the Philippines Diliman, Quezon City 1101, Philippines; 2 Materials Science and Engineering Program, College of Science, University of the Philippines Diliman, Quezon City 1101, Philippines; 3 National Institute of Molecular Biology and Biotechnology, College of Science, National Science Complex, University of the Philippines, Diliman, Quezon City 1101, Philippines; eiprieto@up.edu.ph

**Keywords:** hydrogel, crosslinking, plasma treatment

## Abstract

The use of materials to restore or replace the functions of damaged body parts has been proven historically. Any material can be considered as a biomaterial as long as it performs its biological function and does not cause adverse effects to the host. With the increasing demands for biofunctionality, biomaterials nowadays may not only encompass inertness but also specialized utility towards the target biological application. A hydrogel is a biomaterial with a 3D network made of hydrophilic polymers. It is regarded as one of the earliest biomaterials developed for human use. The preparation of hydrogel is often attributed to the polymerization of monomers or crosslinking of hydrophilic polymers to achieve the desired ability to hold large amounts of aqueous solvents and biological fluids. The generation of hydrogels, however, is shifting towards developing hydrogels through the aid of enabling technologies. This review provides the evolution of hydrogels and the different approaches considered for hydrogel preparation. Further, this review presents the plasma process as an enabling technology for tailoring hydrogel properties. The mechanism of plasma-assisted treatment during hydrogel synthesis and the current use of the plasma-treated hydrogels are also discussed.

## 1. Introduction

The field of biomaterials has been stimulated and accelerated by advances in medicine, biology, chemistry, physics, materials science, and engineering for the last 50–60 years [1]. Even before this interdisciplinary field was recognized, historical records, dated to thousands of years B.C., have shown that biomaterials have been used to restore bodily functions. Examples of early biomaterials include the mineral stone-substitute teeth of a Tlailotlacan woman from 1600 years ago and the Perspex intraocular lens during the 1940s [1,2].

Biomaterials are often made using three general types of materials: metals, ceramics, and polymers [3]. Compared with metals and ceramics, polymeric biomaterials can be easily manufactured with a wide spectrum of physical, mechanical, and chemical properties [3,4,5,6,7]. Because of their versatility, a significant number of biomaterials produced are made from polymers [3]. Among the polymeric materials, hydrogels are among the most promising, able to augment the limitations of traditional biomaterials.

Hydrogels are materials generated from chemical or physical crosslinking of soluble polymers resulting in hydrophilic macromolecular networks. The presence of the crosslinks allows hydrogels to absorb large amounts of water while maintaining the integrity of their three-dimensional (3D) structure [3,8]. Hydrogels are considered ideal for biomedical applications due to their soft tissue-like water content, structure, and flexibility. Their potential use in the fields of medicine and life science, however, is hampered by their low mechanical strength, especially in the swollen state [9]. In addition, their application can be limited due to their crosslinking-dependent dissolution [10], degradation behavior [11], and non-adherent quality [9]. Thus, there is an intensive effort to improve these hydrogel properties for biomedical applications. Novel hydrogel systems and preparation strategies are continuously being sought to overcome these disadvantages.

Plasma technology is usually a fast and efficient process used for material synthesis and surface modification. It is often regarded as a better alternative to chemical and other wet-based treatment where it is more environmentally friendly, can be operated at low temperature (<100 °C), and consumes less chemicals, energy, and time [12,13]. Plasma generates highly reactive species to modify the surface by attaching or etching the functional groups at or near a polymeric surface [14,15,16,17,18,19]. Aside from surface modification, the excited species of plasma can generate radicals that can recombine and induce crosslinking of polymers in the liquid phase [20]. This concept opened the use of plasma technology in hydrogel synthesis.

In this review, we focus on the recent developments of hydrogel biomaterials. Specifically, this review tackles the evolution of hydrogels and how they are prepared in terms of the crosslinking strategies. This review also introduces plasma as an enabling technology utilized for hydrogel synthesis and modification.

## 2. Hydrogels as Biomaterials

Hydrogels were considered as the first biomaterials designed for human body use [21,22]. They belong to the large family of gels that are formed through the crosslinking of hydrophilic natural and/or synthetic polymers. The main distinction of a hydrogel from other types of gel is that the primary fluid that takes up at least 10% of its weight is water [23]. Hydrogels are capable of absorbing water up to a thousand times their dry weight.

**Figure 1:**
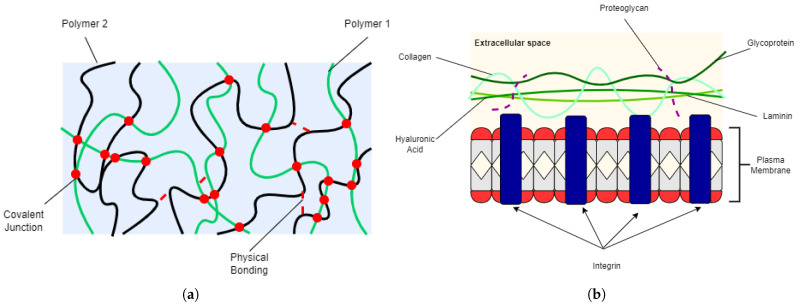
2D illustrations of (**a**) hydrogel and (**b**) ECM.

Table 1 summarizes the general and most important properties of biomaterials [2] for human use. The physical, mechanical, and chemical properties of a biomaterial must be similar or comparable with the body function being replaced or implanted in. Hydrogels provide a framework for tissues to regenerate [24,25] while controlling the diffusion of molecules and cells [26,27]. The water content of hydrogels provide suitable environment for cell seeding and encapsulation [24,28,29] while their porosity allows for the mass transport of gases, nutrients, proteins, cells, and waste products [25]. Despite their soft and rubbery nature, hydrogels have adequate mechanical strength to provide temporary support as scaffolds or implants. The physical and structural properties of a hydrogel resemble the extracellular matrix (ECM) (Figure 1), making them an ideal candidate for biomedical applications.

As one of the earliest biomaterials, the development of hydrogel over the past few years has considered several factors. The network formation through different polymerization techniques, the monomer precursor chosen for hydrogel preparation, and the target properties for the intended biomedical application are among the factors that have evolved in the field of hydrogel biomaterials. The progress in hydrogel studies can be divided into three generations.

## 3. Evolution of Hydrogels

Early work on hydrogels began in 1894, when the use of inorganic salts resulted to a colloidal gel product [30,31]. This established the use of the term ’hydrogel’ to describe a three-dimensional network of hydrophilic natural/synthetic polymers connected chemically or physically [30]. The present definition of hydrogel, however, was based on the pioneering work of Wichterle and Lim [30,32]. Wichterle and Lim, in 1960, developed soft contact lenses using poly (2-hydroxymethyl methacrylate) (pHEMA) gels [21]. This marked the beginning of hydrogel studies for biological applications [33]. PHEMA, which consists of water-swollen crosslinked macromolecular networks, was considered the first synthetic polymer synthesized by DuPont scientists [34].

Since then, hydrogels, as biomaterials, are continuously being improved to meet requirements for the target biomedical applications. The development of hydrogels are distinguished into three generations [32,33,35]. The first generation of hydrogels are chemically crosslinked hydrogels with relatively high swelling and good mechanical strength [32]. Hydrogel synthesis in this period focused on (1) the polymerization of water-soluble monomers and (2) the crosslinking of existing synthetic polymers. The first method employs one of the commonly known chain polymerization techniques which is the free-radical polymerization [36]. Moreover, crosslinking agents were not only used for crosslinking, but also for propagating radical chains to enhance crosslinking.

The second generation of hydrogels were inspired by the works of Kuhn on the conformation of ionizable polymeric molecules [37]. The findings supported the possibility of a reversible contraction on stretched molecules, in the presence of acid or neutral salts. Hence, second-generation hydrogels included those capable of responding to a specific stimuli such as variations in temperature, pH, or ionic concentration in a solution [35]. Main crosslinks for this generation were hydrophobic and ionic interactions [33].

The third generation of hydrogels were inspired by the stimuli-responsiveness of second generation hydrogels. This led to the birth of smart hydrogels that are stimuli-sensitive with tunable physicochemical and mechanical properties [32,35]. Other physical interactions and crosslinking methods such as stereocomplex formation were exploited during this generation [38,39].

The choice of crosslinking technique in preparing hydrogels should always be considered. Crosslinking establishes the network of the hydrogel which determines its eventual properties. These properties can be tailored for a target biomedical application. Moreover, crosslinking in hydrogels can be temporary or permanent, which also affects their intended use.

## 4. Hydrogel Crosslinking Strategies

**Figure 2:**
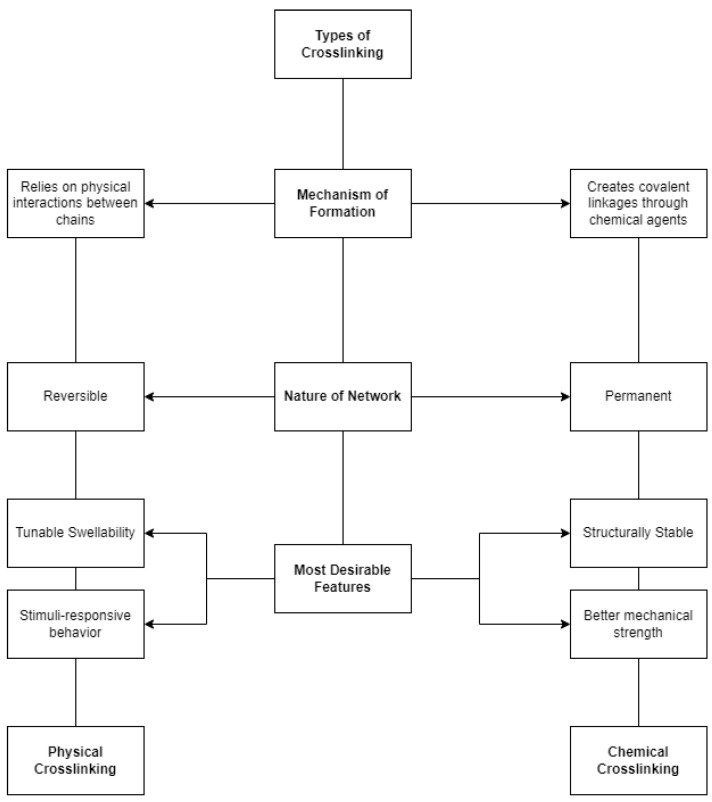
Comparison between physical and chemical crosslinking.

The term crosslink refers to the covalent or secondary connection points of several chains [40]. It can be a covalent link that is a small chemical bridge between carbon atoms, or a junction created by crystallites or secondary interactions. The integrity of a hydrogel is highly associated with the presence of crosslinks in its structure [3,41]. Thus, hydrogels are distinguished according to their crosslinking network. Hydrogels can either be prepared by physical or chemical crosslinking. Comparison between the two crosslinking types in terms of the mechanism of formation, nature of the network, and desirable features are shown in Figure 2.

Chemically crosslinked hydrogels have more tunable physicochemical properties than the physically crosslinked hydrogels [42]. Aside from that, they also have higher crosslinking density, resulting in their better mechanical strength. With this, chemically crosslinked hydrogels may be favored for biomedical applications. Having a high crosslinking density, however, is not often desirable as it can also result in the lowering of swelling capability and pore size of a hydrogel. The changes in these physical properties can affect how biomolecules and cells diffuse in the hydrogel [43]. Thus, crosslinking must be modified to improve the biological interaction of the hydrogel [44]. The different types of physical and chemical crosslinking are briefly discussed in the following subsections.

### 4.1. Physical Crosslinking in Hydrogels

#### 4.1.1. Hydrogen Bonding

Crosslinking via hydrogen bonding is possible with polymers containing polar functional groups such as hydroxyl, acylamino, and carboxyl groups [45]. When carboxyl groups protonate, hydrogen bonding can occur with other polymers containing electron-deficient hydrogen atoms [46]. The network of poly(ethylene glycol) (PEG) can act as template for the polymerization of poly(acrylic acid) (PAA) and the interpenetrating network (IPN) can be formed through hydrogen bonding with acrylic acid monomers [47]. The hydrogen bonding for this network usually occurs at low pH where carboxyl groups protonate [48]. As a result, PEG-PAA hydrogel can be used as an artificial corneal implant with a transparent appearance and safe to use up to 6 months.

Hydrogen bonding in hydrogels can also be in the form of (1) hierarchical hydrogen bond system [49] or (2) two-step hydrogen-bonded densified network [50]. These strategies were used to fabricate N,N-dimethylacrylamides (DMAA)—acrylic acid (AA) and densified hydrogen-bonded iron-chitosan-poly(acrylic acid) (DHB-Fe/Cs/PAA) hydrogels with high stretchability, self-healing property, and fatigue resistance features.

#### 4.1.2. Coacervation Process

**Figure 3:**
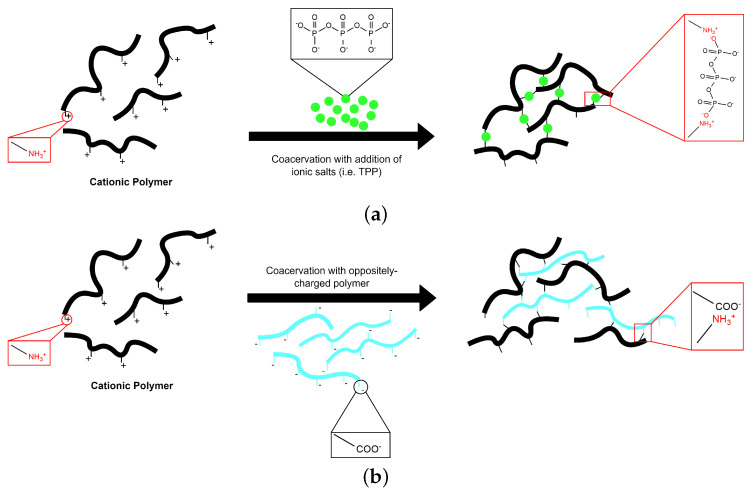
Schematic illustration of hydrogel formation by (**a**) simple and (**b**) complex coacervation processes.

The term coacervation is used to denote a phase-separation process in colloidal chemistry. This process is induced by a modification in the media environment (pH, ionic strength, temperature, solubility) under controlled conditions. Coacervation involves two phases [51]. The coacervate phase is rich in colloid while the equilibrium phase contains little amounts of colloid [52]. Depending on the involved polymer systems and phase separation mechanism, the coacervation processes can be differentiated into two types: the simple and complex coacervation [51,53] (Figure 3). 

The simple coacervation process involves a single polymer being coacervated upon the addition of a salt or a coacervation agent. The presence of ionic residues (e.g., di- or trivalent counter ions) creates charged interactions that influence the crosslinking in the solution [46,54]. Ionic agents such as calcium (Ca${}^{2+}$) and tripolyphosphate (TPP) have been used to gelate alginate [55] and chitosan [56], respectively. The electrostatic attraction between these polyelectrolyte complexes results in an improved protein encapsulation and pH-sensitive controlled release for drug delivery system.

For complex coacervation, there should be at least two oppositely charged polyelectrolytes (usually proteins and polysaccharides) involved. This interaction results to the formation of soluble and insoluble phases, depending on the concentration and pH of the respective solutions. The glucoronic acid and pyruvate chains of xantham gum were shown to interact with the amino groups of chitosan to form a complex-coacervated hydrogel [57,58,59]. The ionic groups present in the xantham–chitosan network can be tuned by varying the concentration of both polyelectrolyte. The resulting hydrogel has significant swelling dynamics for an intestinal drug delivery system [60].

#### 4.1.3. Heating or Cooling a Polymer Solution

Hydrogels can also be formed through heating or cooling. This is possible especially with polymers that have helices, helix-like formation, and junction zones present in their structure [46,48]. Carrageenan–gelatin hydrogels are prepared by mixing hot stock solutions of each precursors at 40 °C in different weight ratios [61]. The carrageenan–gelatin complex is initially formed at 40 °C due to its random coil and hydrophobic interactions. At lower temperatures, the hydrogel stabilizes due to electrostatic interaction. This is manifested when the complex transforms into a helix conformation [62]. Another hydrogel produced using this approach is the PEG and poly(lactic acid) (PLA) hydrogel. PEG/PLA block copolymers are synthesized as physical hydrogels by mixing solutions of each polymer, first cooled to 10 °C, with varying mass ratios to have an enantiomeric mixture [63]. The mixture is then heated from 10 to 70 °C and gelation is initiated once temperature has reached equilibrium. Both hydrogels are used for drug delivery applications. The porosity and thermoresponsive behavior of gelatin–carrageenan controls the regulation of drug release [61]. The PEG/PLA hyrogels, on the other hand, allow the encapsulation of hydrophobic drugs [61,63].

#### 4.1.4. Crosslinking by Crystallization

**Figure 4:**
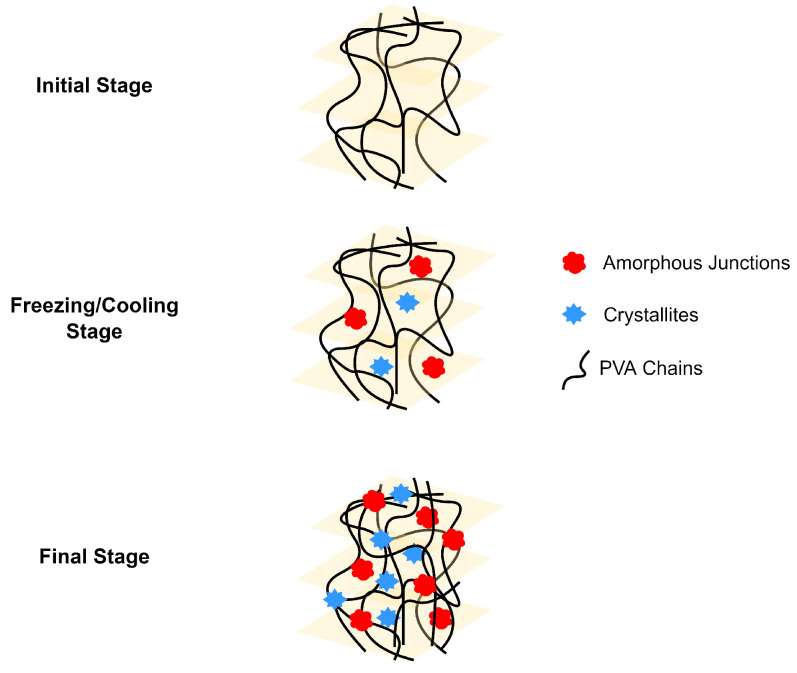
Representation of PVA gelation by freeze/thaw cycle.

The crystallites in a polymer chain can act as physical crosslink sites in the network, leading to the formation of a hydrogel [41]. The crosslinking, in this approach, can be carried out in a temperature within the freezing range. Poly(vinyl alcohol) (PVA) is a well-known water-soluble polymer that can be prepared by physical crosslinking. Freeze/thaw cycle method has been used to prepare PVA physical gels as it can modify the polymerization of PVA [64,65,66]. The mechanism for the physical crosslinking of PVA is shown in Figure 4. Gelation of PVA initially takes place by spinodal liquid–liquid phase separation, which would cause the formation of a porous network structure [64]. The freeze and thaw cycles creates amorphous and crystalline regions in the PVA hydrogel. The amorphous regions are created when the free water molecules occupy the junction or voids in the phase separation. Crystalline regions, on the other hand, are present due to the aggregation of crystallites. These regions both act as crosslinking points for the PVA hydrogel. The resulting PVA hydrogels have a thermoreversible sol-gel transition with mechanical and thermal properties dependent on the number of freeze/thaw cycles [65,66].

### 4.2. Chemical Crosslinking in Hydrogels

#### 4.2.1. Free Radical Polymerization

The free radical polymerization (FRP) is a chain-growth polymerization technique in which initiators create free radicals by either homolytic dissociation or redox reaction [67]. The carbon–carbon double bonds of monomers are usually the active sites for free radicals to induce chain propagation. The chain then terminates when the propagating radicals react by combination, disproportionation, and transfer. With this, the FRP approach is the most common chemical crosslinking route for hydrogel preparation [2,44]. This technique involves rapid propagation of the active sites from monomers, resulting in the fast synthesis of hydrogel networks. Conversely, the hydrogel produced in this approach is heterogenous. In this regard, hydrogels are prepared in a non-controlled manner where crosslinking is not consistent and double bonds and side chains can be inaccessible for further functionalization and polymerization [68]. Hydrogels based on monomers such as acrylates, vinyl lactams, and amides are usually prepared by FRP [44,46]. Examples of chemically crosslinked hydrogels and their characteristics are shown in Table 2.

#### 4.2.2. Photopolymerization

Photopolymerization enables the in situ formation of crosslinked networks [67]. It provides a unique way of forming gels in a fast and controllable manner [74]. In this technique, visible/UV light is used to interact with light-sensitive compounds, called photoinitiators, and convert liquid monomer or macromer to a hydrogel by FRP. It has several advantages over conventional polymerization techniques, such as spatial and temporal control over polymerization, fast curing rates at room or physiological temperatures, and minimal heat production [27,75]. The mechanism for photoinitiation depends on the photolysis processes: photo-cleavage, hydrogen abstraction, and cationic photopolymerization [27,67].

The structure of the photoinitiated chitosan/PAA polyelectrolyte complex is created from the intermolecular association of chitosan amino groups and PAA carboxyl groups with [2,2${}^{\prime }$-azobis-(2-amidinopropane) dihydrochloride] as a photoinitator and UV irradiation as crosslinker [76]. PEG-fibrinogen hydrogels, on the other hand, can be produced by photocrosslinking using Irgacure 2959, Irgacure 184, and Irgacure 651 photoinitiators [77]. The stability of photocrosslinked hydrogels depends on the water content, photoinitiator concentration, and irradiation intensity. These parameters may affect the swelling behavior, elasticity, and cell viability of the hydrogels.

#### 4.2.3. Crosslinking Induced by Enzymatic Reactions

Enzymatic crosslinking allows the formation of strong covalent bonds and rapid gelation (<10 min) under physiological conditions [41]. Similar to photopolymerization, enzymatic crosslinking is considered an attractive method for in situ hydrogel formation. Pullulan-based injectable hydrogel scaffold is commonly prepared through UV crosslinking followed by curing at 50 to 60 °C [78,79]. This approach, however, is deemed too harsh for an in situ injectable hydrogel for tissue engineering [80]. The enzymatic crosslinking therefore is preferrable in such applications as it can perform crosslinking at around 36.1 to 37.2 °C. Aside from the fast gelation, enzymatic crosslinking also offers an adjustable mechanical property and controllable degradation while maintaining the cyto- and tissue compatibility of the hydrogel. Examples of enzymatically-crosslinked hydrogels are summarized in Table 3.

The enzymes typically used in this crosslinking approach include horseradish peroxidase (HRP), tyrosinase, transglutaminase, lysil oxidase, and plasma amine oxidase [54,80,81,82,83,84,85]. HRP is the most widely used enzyme due to its cheap availability, fast gelation, and tunable crosslinking density [82,84,85]. It acts as an anaerobic oxidase where the valency of peroxidase iron changes by going through a ferric–ferrous cycle [84]. Its oxidase reaction can be modulated with hydrogen peroxide (H${}_{2}$O${}_{2}$). HRP-mediated crosslinked hydrogels are synthesized using polymers containing phenols, phenylamines, indoles, and sulfonates. The covalent bonds are formed between hydroxyphenol groups [80,84]. HRP, however, exhibits very poor enzymatic activity and catalytic stability under aggressive processing conditions [85]. Cytotoxicity may also be induced through radical formation at high concentrations of HRP and H${}_{2}$O${}_{2}$ [81,82].

**Table 3:** Examples of enzymatically crosslinked hydrogels and their applications.

Hydrogel	Application	Reference
Carboxymethylated pullulanchondroitin sulfate	Cartilage scaffold	[80]
Polyphenol-epigallocatechin gallates	Tissue adhesive	[81]
Tyramine-silk fibroin	Cell delivery	[82]
Agarose-chitosan	Biocatalysis	[83,85]

#### 4.2.4. Crosslinking by “Click Chemistry”

Click chemistry, a term coined by Barry Sharpless, represents a group of reactions that are fast, versatile, purifiable, and with high product yields [86]. The process was formed to mimic the aldol condensation of natural materials. Natural products tend to link through the C-C bonds in their backbones [87,88]. Its synthetic reproduction requires a high thermodynamic driving force. Click reactions address this concern by combining a C atom to a heteroatom X which is abundant in polysaccharides. As a result, a C-X-C bridge is formed, instead of a C-C bond, to link proteins, nucleic acids, and/or polysaccharides. The hydrogel crosslinking based on click chemistry includes the following: Diels–Alder, Schiff base, oxime, Michael-type addition, and boronate ester. Diels–Alder addition reaction produces hydrogel by cycloaddition of diene and/or dienophile to induce complementary moiety for crosslinking [89,90]. Schiff base reaction creates an imine (C=N) linkage between the amino and aldehyde groups of two polymers [91,92]. The oxime crosslinking, on the other hand, forms a hydrogel by reacting an aminooxyl/hydroxylamine group and an aldehyde/ketone [93,94]. Michael addition involves a reaction between nucleophiles called Michael donors and activated electrophilic olefins/alkynes (Michael acceptors) [95,96]. Lastly, the boronate ester bond is an integral part of the dynamic covalent bond (DCB)-based hydrogels where it is formed through the condensation reaction between boronic acid and 1,2- or 1,3-diols [97,98].

**Figure 5:**
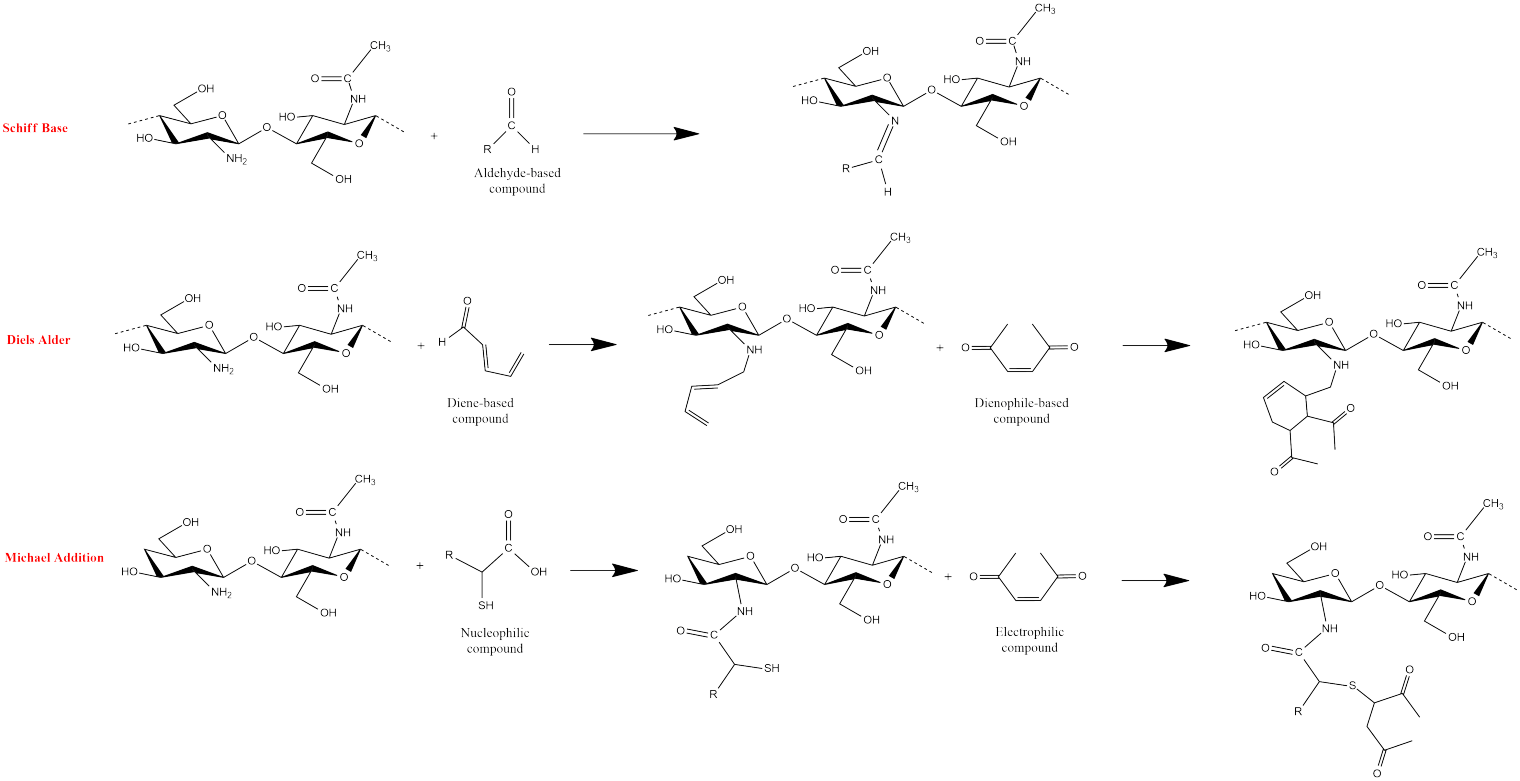
Schematic representation for the formation of chitosan-based hydrogel by click reactions.

Schematic formation of chitosan-based hydrogels through different click chemistry routes is shown in Figure 5. A pH-responsive N-succinyl-chitosan (NSC) semi-interpenetrating network (semi-IPN) hydrogel was synthesized through a Schiff base mechanism and with glutaraldehyde crosslinker. The semi-IPN NSC hydrogel showed potential as targeted oral drug delivery carrier due to its rapid release of 5-fluorouracil (FU) drug at pH 7.4 [91]. Hybrid chitosan-gelatin hydrogel was prepared by Diels–Alder reaction. Methyl furan and maleimide were utilized to crosslink the two polymers [89]. The synthesized hybrid hydrogel provided self-healing features and applicability for 3D bioprinting which is useful in the fields of tissue engineering and advanced biological studies. Lastly, in a Michael addition reaction, chitosan was first modified by thiolation to fabricate a thiol-modified chitosan (CsSH) hydrogel for biomedical application. A tunable rheological and swelling behavior, depending on the amount of the bismaleimide (BMI) crosslinker, was demonstrated. The in vitro degradability of CsSH hydrogels against lysozyme enzymes depends on the amount of the BMI crosslinker [95].

#### 4.2.5. Grafting

**Figure 6:**
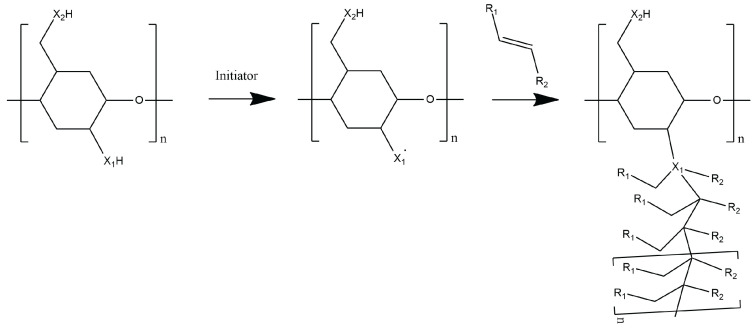
General scheme of the grafting process.

Graft polymerization is a versatile technique of adding desirable functional groups into the polymer backbone, producing a new hydrogel with tailored properties [99]. Grafting involves the generation of active sites by abstraction of hydrogen atom from the polymer backbone. This generates macroradicals where desired monomers can attach to produce a graft copolymer [100,101]. The general reaction mechanism for grafting is shown in Figure 6. The properties of the resulting graft copolymers are controlled by the molecular structure, length, and number of the side chains [102]. Grafting can be achieved through chemical, radiation, photochemical, plasma-induced and enzymatic methods. In this review, the chemical, radiation, and plasma-induced grafting are presented.

Chemical grafting is based on FRP and is the most widely used grafting method [48,103]. It utilizes initiators that can either generate free radicals or form ionic centers (cationic or anionic) to commence the grafting process [99]. Initiator systems used for free radical-initiated grafting include ferrous ammonium sulfate (FAS), ceric ammonium nitrate (CAN), potassium diperiodatocuprate (III), potassium persulfate (KPS), thiocarbonationpotassium bromate (TCPB), and ammonium persulfate (APS), while ionic-induced grafting can be triggered by sodium methoxide, alkyl aluminum, tertiary butyl phosphazene, and Fe${}^{2+}$-H${}_{2}$O${}_{2}$ [103,104,105,106]. Studies that have produced hydrogels using this technique include grafting vinyl monomers onto polysaccharides such as starch [101,105], alginate [107,108], cellulose [109,110], and chitosan [111,112]. The grafting of monomers such as AA, HEMA, and butyl methacrylate (BMA) onto the starch backbone was shown to influence the swelling and drug release mechanism of the hydrogel, resulting in a potential intestinal-targeted drug carrier [113].

Grafting through irradiation creates free radicals by inducing homolytic fission to the macromolecules [48,103,104]. Radiation sources for this grafting method utilize microwave (MW), UV, and gamma irradiation [48]. In this method, the polymer backbone is irradiated. The resulting products have narrower molecular distribution compared to the conventional chemical grafting. The direct radiation exposure of polymers, however, may lead to chain scission of the base polymer. This degradation may cause the formation of a block copolymer instead of a graft polymer [103]. Radiation grafting of silicone rubber with N-vynyl pyrrolidone (NVP) resulted in an improved thrombo-resistance of silicone rubber when a grafting ratio of around 30–35% was achieved [114]. The hydration behavior of polyester-urethane improved after radiation grafting. This makes the hydrogel viable for biomedical application. The poly(2-hydroxyethyl methacrylate) (HEMA) was grafted onto the polyurethane surface causing the restructure of polymer chains: polar groups inside the urethane substracte and aliphatic carbon near the surface [115].

Both crosslinking types have their own advantages and disadvantages. Hence, there is a continuous effort in hydrogel studies to seek a novel and/or different approach. An alternative method for hydrogel synthesis may be explored where the complexity of the process, including the use of chemicals and high energy operations, is limited and at the same time, economical and safe in achieving tunable functionalities without compromising the structural integrity of hydrogels for biomedical application. The use of plasma technology for hydrogel synthesis will be thoroughly discussed in the succeeding sections. The interaction of plasma with the surface as well as the polymerization in liquid phases will be discussed. The biomedical applications of plasma-assisted fabrication of hydrogels will be presented.

## 5. Plasma–Material Interactions

**Figure 7:**
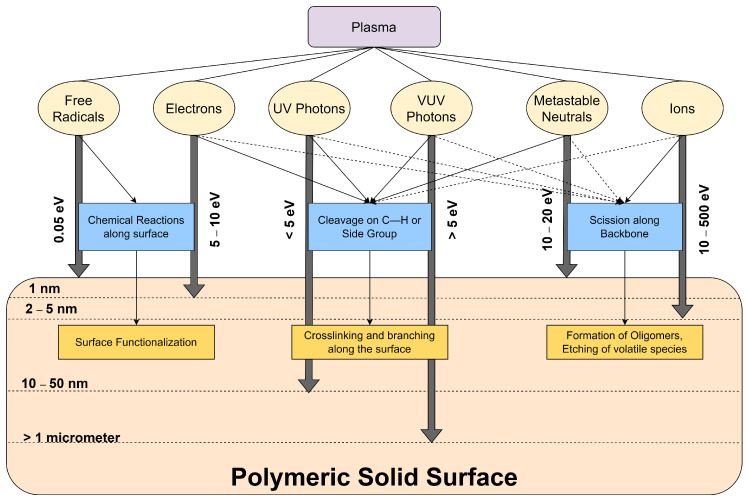
Interactions of different plasma species on a polymeric material.

Plasma can be classified as a non-equilibrium state of gas. When its elementary particles (ions and electrons) interacts with a surface, the plasma may find suitability in tailoring the surface properties of materials such as polymers [116]. Neutral particles, vacuum ultraviolet (VUV) and UV photons are also present in the discharge. As shown in Figure 7, different interactions may occur once these highly reactive species reach the polymer surface. The exposed particles and photons in the polymer surface can be modulated by factors such as the plasma operating parameters, polymer substrate, gas chemistry, and the reactor design [117]. The excited plasma species continuously bombard the polymer surface, resulting to the change in the chemistry and characteristics of the surface structure and/or morphology [118,119]. The plasma and surface interaction may result to different processes such as functionalization, crosslinking, and etching. These processes may occur in isolation or in synergistic combination and they all affect the adhesion behavior of the polymer with other substrates [117].

**Figure 8:**
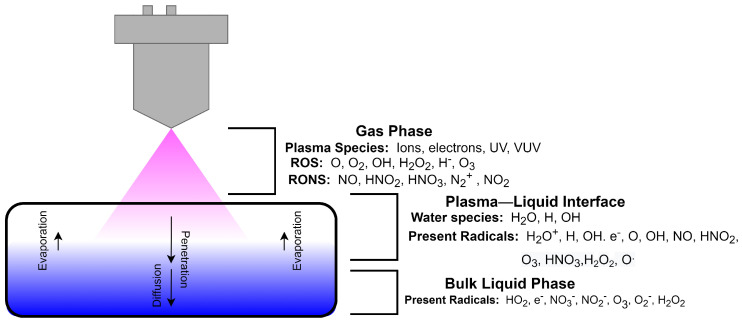
Schematic diagram of plasma interacting with a liquid.

Interactions between plasma and liquid solutions, on the other hand, remain largely unexplored, complex, and deemed challenging. Figure 8 shows a schematic representation of the interactions in a plasma–liquid system. When a liquid sample is exposed to plasma, the interaction can be investigated under these phases: gas phase, plasma–liquid interface, and bulk liquid phase. The gas phase is composed of the reactive species generated by the plasma shown in Figure 7. In this region, the plasma species interact with the ambient air, resulting to the formation of additional reactive oxygen and nitrogen species (RONS). The generated RONS approach the plasma–liquid interface where these species produce additional radicals with the water vapor. The unreacted RONS then penetrate the liquid phase by various transfer process such as collision, diffusion, absorption, and chemical transfer [120]. Due to their highly energetic nature, electrons can leave the bulk plasma and induce energy-dependent reactions. With the presence of energetic electrons, additional interactions can occur with the chemical species in the bulk liquid phase [121]. The newly-formed radicals in the plasma–liquid interface will tend to remain in the bulk liquid phase to initiate post-discharge reactions within the liquid. The excess unreacted and unbound electrons may also create additional reactions in the liquid phase.

## 6. Surface Modification by Plasma Technology

**Figure 9:**
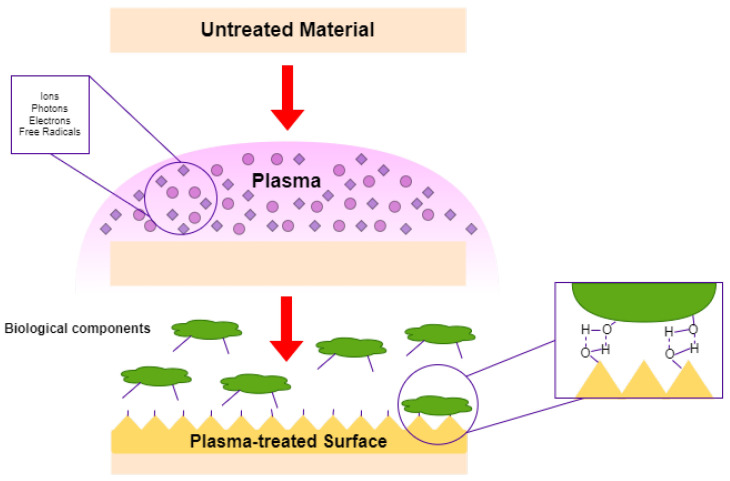
Schematic of plasma modification of surfaces.

Most of the biomedical studies using plasma treatment focused on functionalizing the surface of biomaterials [12,15,122,123,124,125,126,127]. As shown in Figure 9, plasma treatment creates a biomimetic microenvironment on the biomaterial surface. Biomimetic layers ideally embody essential features of natural tissues such as hydrophilicity, lipophilicity, porosity, and self assembly [128,129,130,131]. Innovative technologies in chemistry and processing have been utilized to achieve biomimetic scaffolds or membranes capable of mimicking the native ECM and other functionalities such as regulation, proliferation, and other cellular interactions [132,133]. The application of plasma treatment is a common approach to improve the surface properties of biomaterials while retaining the adequate bulk properties, in terms of physical, mechanical, and thermal stability [130,134,135,136].

Plasma treatment is a novel approach to produce biomimetic membranes. The treatment works on different atmospheres to tailor the surface functionality [123]. The injected power, gas admixture, and process duration are considered the most critical plasma treatment parameters [12,15,126] to improve the biological responses of biomaterials such as protein adsorption [127], and cell [123] and lipid [126] adhesion.

Plasma technology is regarded as an environmentally friendly process with short processing time and low cost, compared to the wet-based finishing processes [117,124,137]. Moreover, it can selectively treat depths of up to few nanometers of the polymer surface [12] to meet satisfactory biological response with surface-sensitive biological systems [124,138]. Other advantages of plasma modification include deposition of crosslinked films on complex geometries, formation of multilayer films, rapid process, sterility, and ease of scaling up the system [139].

## 7. Early Use of Plasma Treatment in Liquid Solutions

**Figure 10:**
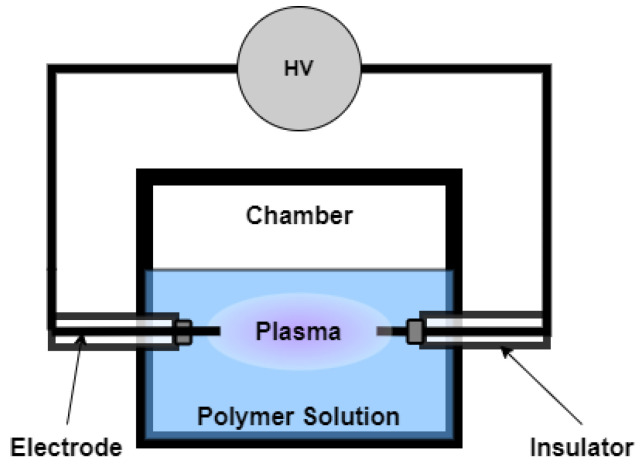
Schematic diagram of the plasma system used for wastewater treatment.

The use of plasma system in liquid solutions has started in wastewater treatment applications [140,141]. Chemical oxidation process is one of the main solutions for wastewater treatment. However, it can also form partially toxic chemical intermediates and products. An alternative solution presented for water treatment and purification is the use of an electrical discharge-based system, as shown in Figure 10 [140]. In this system, highly active species such as H${}_{2}$O${}_{2}$, O, OH, HO${}_{2}$, O${}_{3}$*, N${}_{2}$*, e${}^{-}$, O${}_{2}^{-}$, O${}^{-}$, and O${}_{2}^{+}$ can be produced. A bipolar pulsed power supply was able to ignite an electrical discharge in the aqueous solution. This alternative, however, used a relatively high voltage power supply (at most 1 kV) to decompose the organic compound wastes. Eventually, a plasma system with low voltage operation (100–300 V) was successfully utilized to treat wastewater [141].

The use of plasma in treating liquid samples was then carried out in modifying natural polymers in solution [16,142]. A glow discharge system was used to modify a starch slurry [142]. Chemical analysis on the starch slurry revealed that the -OH functional group of the biopolymer decreased and no C=O bonds were formed. With this observation, it was suggested that the starch underwent crosslinking instead of oxidation along the -OH bond. For the case of the gelatin solution, the exposure of the polymer solution to plasma showed generation of free radicals such as -OH. Prolonged plasma exposure (>10 min), however, could lead to the slow generation of free radicals and progression to crosslinking [16].

## 8. Mechanisms in a Plasma-Assisted Hydrogel Synthesis

**Figure 11:**
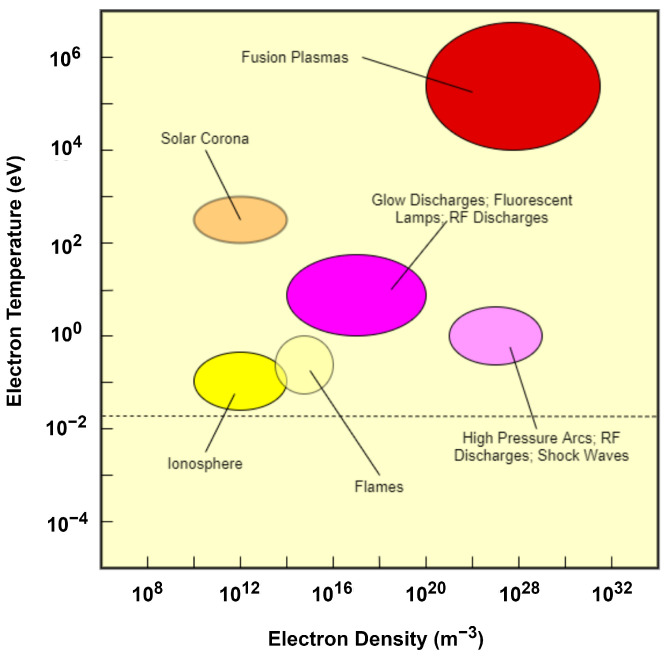
Classification of plasma in terms of electron temperature and electron density.

At present, mechanism and effects of plasma to liquid solutions have not been fully established. Despite this, there are studies that have included plasma treatment for hydrogel synthesis. Specifically, atmospheric pressure plasma (APP) has already been investigated for hydrogel fabrication. APP lies along the glow discharge and arc plasmas [143], as shown in Figure 11. APPs, therefore, have high electron density even at low temperatures. APPs can be ignited by a 100–250 V power source and they have high plasma chemical activity even at standard temperature and pressure [144,145,146]. The generation of reactive species, without solely relying on chemicals and high energy such as radiation, has made plasma treatment a viable candidate in fabricating hydrogels. Plasma treatment can be vital in the initiation or crosslinking stage of the hydrogel synthesis.

### 8.1. Plasma-Initiated Polymerization

**Figure 12:**
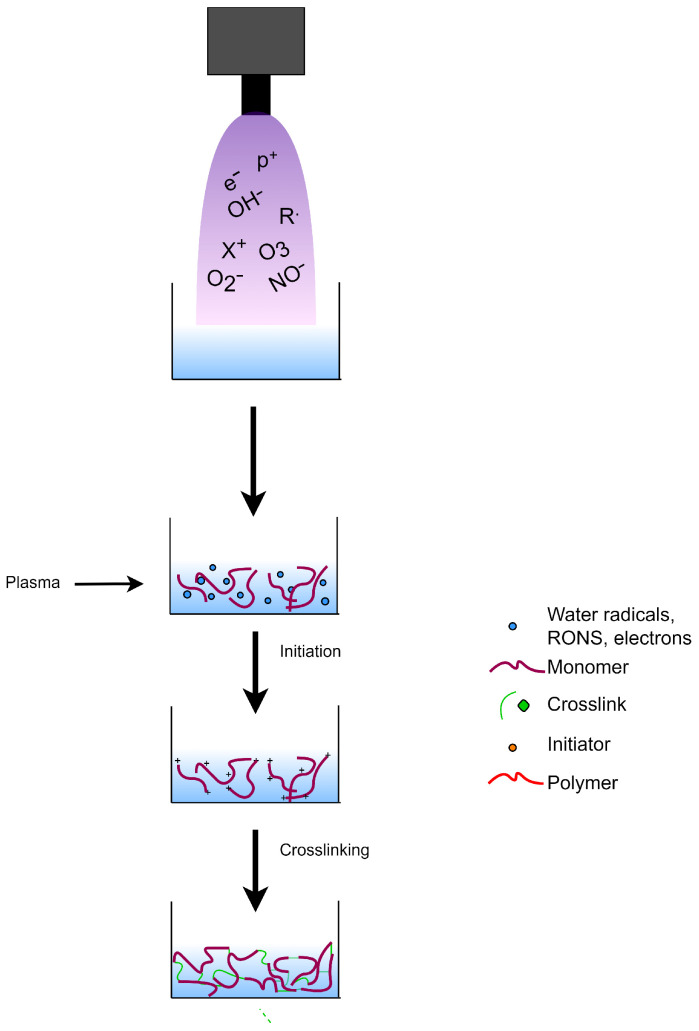
Use of plasma treatment as initiator during hydrogel synthesis.

Considering its ability to modify polymeric solutions, glow discharge electrolysis plasma (GDEP) was carried out to induce polymerization reactions in hydrogels. The setup for GDEP is not expensive, unlike those used for UV-curing and radiation polymerization. The abundant source of free radicals in the aqueous solution makes GDEP a good strategy for copolymerization [147]. The resulting free radicals in the aqueous medium may act as initiators to commence the polymerization process for the hydrogel synthesis. The mechanism for plasma-initiated polymerization for hydrogel synthesis is shown in Figure 12.

Copolymers of poly(vinylpyrrolidone) (PVP)/AA [147], and AA/carboxymethylcellulose (CMC) [148] were fabricated as superabsorbent hydrogels using GDEP. Depending on the synthesis conditions (discharge voltage, time, mass ratios, and crosslinker), the hydrogels can be tuned with reversible swelling-deswelling and stimuli-responsive behavior. GDEP-synthesized cellulose-based ionic hydrogel showed multiresponsive behavior to the change of pH and ionic species and concentration [149]. The acidic–neutral solutions influenced the swelling-deswelling behavior of the ionic hydrogel, while volume shrinkage and decreased swelling ratio were observed in the presence of Zn${}^{+2}$ and Fe${}^{+3}$ ions. Hemicellulose-based hydrogels were also successfully fabricated using a GDEP-initiated technique. The resulting multifunctional hydrogel can either achieve a dual pH/temperature sensitive behavior [150] or thermoreversible upconversion luminiscence (UL) properties [151].

### 8.2. Plasma-Induced Crosslinking

**Figure 13:**
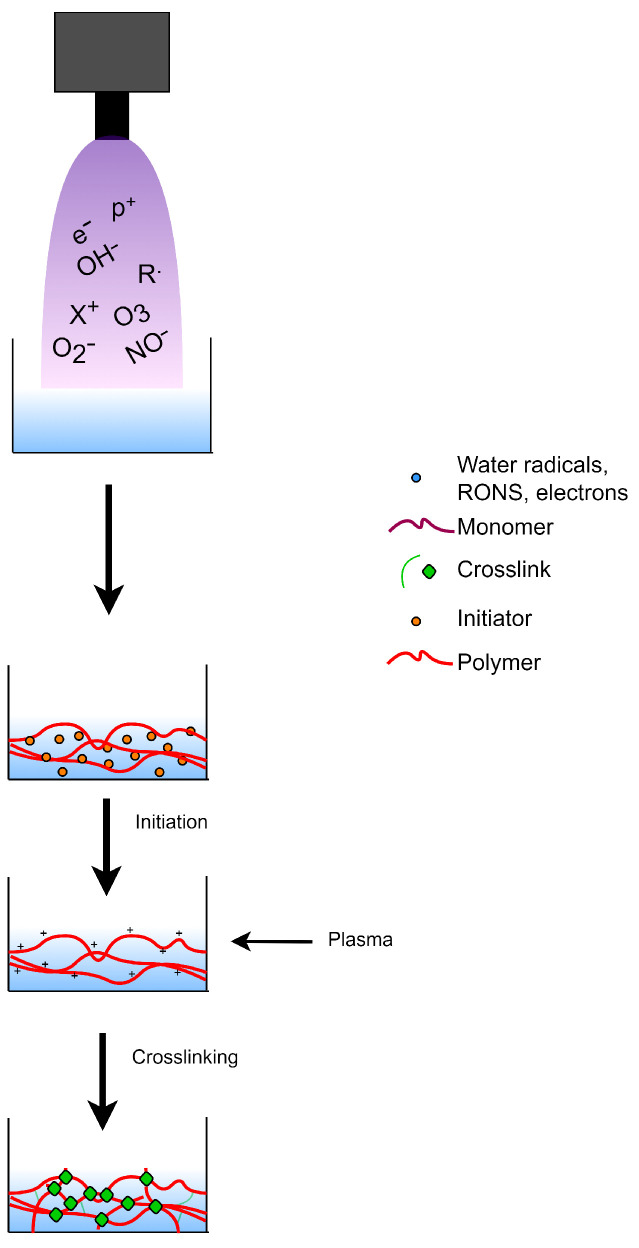
Crosslinking by plasma treatment during hydrogel synthesis.

**Figure 14:**
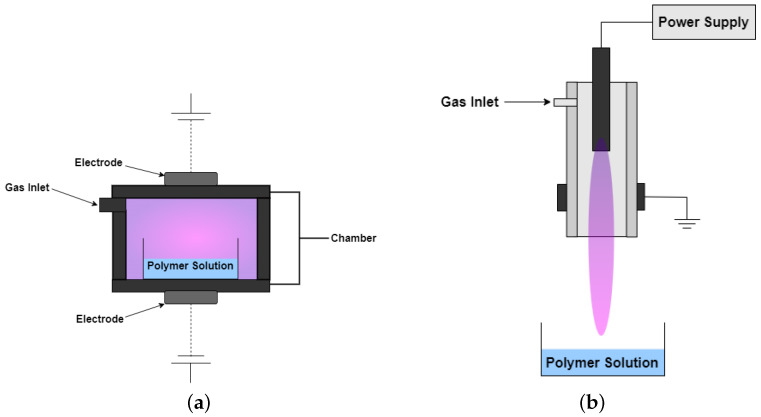
Schematic of (**a**) DBD and (**b**) plasma jet used for hydrogel synthesis.

The generated RONS from plasma may also facilitate crosslinking during hydrogel synthesis as shown in Figure 13. The APP systems dielectric barrier discharge (DBD) and jet, shown in Figure 14, have already been utilized to prepare chitosan-based hydrogels. Chitosan gelation by atmospheric DBD showed that the hydrogel viscosity was dependent on the plasma treatment and chitosan concentration [152]. Different plasma reactivity was observed at different chitosan concentrations. Oxidation and fragmentation of chitosan was observed at low concentration, resulting to a decrease in viscosity. At higher concentration, there was an observed increase in viscosity which was attributed to the possible formation of higher chitosan molecular weight conjugates. Chitosan-acrylic acid blend hydrogel was prepared using an APP jet system. The polymer network was governed by the electrostatic interaction of the chitosan NH${}_{3}^{+}$ and acrylic acid COO${}^{-}$. With plasma treatment, another linkage may have been established through the C-O bridge. A multivariate analysis approach further confirmed that the change observed along the C=O bond and C-O linkage of the hydrogel is significant at higher chitosan concentration (2.5 wt%) than lower chitosan concentration [153]. The viability of APP treatment as a precrosslinking step was also observed with a poly(ethylene oxide) (PEO) model. With APP treatment, the hydrogel network was established with partial covalent crosslinking (formation of C=O bonds) and aggregation of micelles.This results in the decrease in the molar mass and increase in the viscosity. The precrosslinking step increased the extent of crosslinking of PEO hydrogels after they were exposed to the photo-irradiation (2nd crosslinking). The APP-treated hydrogels have twice the elastic modulus of the pristine hydrogels [154].

## 9. Roles of Plasma-Assisted Hydrogel Biomaterials

For the past few years, plasma treatment has been realized as an efficient technology to tailor the surface properties of biomaterials [12,15,122,123,124,125,126,127]. Plasma allows the creation of biomimetic microenvironment suitable for cells to perform their physiological activity. Several studies have already employed plasma treatment on hydrogels to improve their functionalization in the biomedical field. Table 4 enumerates representative works that use plasma to synthesize or modify hydrogels. The use of plasma in facilitating the synthesis of hydrogels paved another way of improving or introducing desirable properties to hydrogels.

Hydrogels such as gelatin-graphene oxide (GO) are established by H-bonding. The use of a microplasma tool produced more radicals, which eventually improved the crosslinking density of the composite hydrogel. Aside from being a stronger and viscoelastic hydrogel, the gel-GO hydrogel has better porosity, hydrophilicity, biocompatibility and swelling behavior compared to a genipin-crosslinked gelatin hydrogel. This results to a more densed crosslinked network gelatin-GO hydrogels [174,175].

Utilizing plasma in liquid phases also offer intra- and intercellular processes which are suitable for biomedical applications. The RONS generated during the plasma-liquid interaction can be stored in hydrogels. This in turn allows hydrogel to be a carrier of RONS like H${}_{2}$O${}_{2}$, which is known to be a key component for wound healing, antimicrobial, and anti-cancer properties of non-thermal plasmas [177]. These RONS-carrier hydrogels can emerge as new alternatives for plasma-based therapies.

Aside from being an RONS carrier, hydrogel may also be used as template for processing nanoparticles (NPs). NPs such as Fe${}_{3}$O${}_{4}$ can be synthesized without yielding negligible effects in the chemical bonding of PNIPAm hydrogel. This results in a thermoreversible hydrogel with magnetic behavior [176]. Antibacterial NPs such as gold and silver may also be processed in hydrogels where polymers such as PVA functions as a capping agent to prevent agglomeration. Physicochemical properties of the PVA hydrogel were not compromised while introducing antibacterial nanoparticles [168].

## 10. Perspectives

### 10.1. Future Direction of Hydrogels

In the past few decades, commercially available biomaterials have exhibited inertness. This property enabled any material to be utilized as a replacement for a damaged human body part without adverse effects. Bioinertness, however, confines the compatibility of materials such that inert materials can not support regeneration, growth, and other functions of the body. Thus, aside from biocompatibility, biofunctionality has become a desirable property for biomaterials.

**Figure 15:**
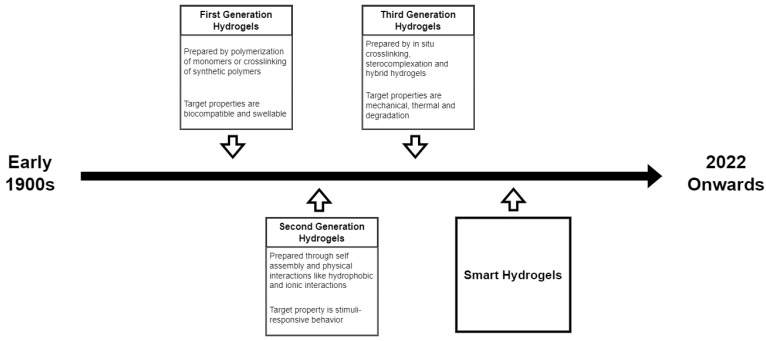
Evolution of hydrogels in terms of preparation and target properties.

There were conditions being examined prior to the use of hydrogels as a biomaterial. Hydrogels went through several stages of development, as shown in Figure 15. Hydrogels were initially developed through conventional polymerization routes where the process involves different chemicals for initiation and crosslinking. Despite the stability of the fabricated hydrogels at swollen state, their biomedical applications were limited due to the toxicity of the chemical agents used. With more natural and synthetic polymers being exploited, strategies shifted in maximizing the ability of hydrogen bonds and other side chains to create the entanglements of the hydrogel network. This resulted in physically entangled hydrogels with an adjustable swelling behavior, depending on the stimulus. The physical entanglements, however, are relatively weak compared to the covalent linkages produced in traditional polymerizations. With the aid of technological advancements, hydrogel matrices can now be composed of natural and synthetic polymers held together by a combination of chemical or physical crosslinking. The development of hydrogels is usually driven by better understanding of the fundamentals, advances in techniques, availability of technologies, and the clamor for new and improved solutions.

Smart hydrogels continue to draw attention due to its responsiveness to different environmental stimuli. This predictable responses will provide tailored experiences to the end-users. Future research can focus on hydrogels that can be programmed to be responsive to multiple stimuli such as biological, physical, and chemical signals. Biomimetic properties should also become intrinsic in synthetic hydrogels. Hence, there should be continuous effort in integrating engineering approaches in developing hydrogel biomaterials. Future studies must find a way to come up with novel and innovative hydrogels with structural components that can be compatible and easily integrated with the proteins and other biological components of the body. In this way, smart hydrogels are on the path of being utilized in a wider range of applications including targeted drug delivery, shape memory implants, biosensors, tissue engineering, and regenerative medicine.

### 10.2. Plasma-Synthesized Hydrogels

Motivated by the need to address the issues raised in past hydrogel generations, the use of an enabling technology such as plasma can be a viable alternative for hydrogel synthesis. Plasma treatment has long been exploited as a surface modification option for biomedical applications. The surface functionality of biomaterials can be efficiently improved without affecting the bulk properties significantly. Moreover, the field of plasma medicine has slowly gained recognition due to the ability of plasma to produce RONS. These species provide beneficial effects in applications such as wound healing and cell therapies. Plasma can provide a controllable highly reactive environment that can be used to tailor specific properties of hydrogels.

Since it can generate RONS, plasma may be able to facilitate hydrogel synthesis either through the initiation step or during the crosslinking process. In these synthesis routes, hydrogel biomaterials may offer essential advantages such as simplicity, time efficiency, and minimal-to-no use of chemicals and high energy. The resulting hydrogel may not only have networks established by physical and chemical crosslinks, but it can also have a RONS-enriched environment. More studies should be conducted to fully appreciate the influence of direct and indirect plasma treatment in the synthesis of hydrogels.

## 11. Conclusions

This review focused on the evolution and development of hydrogels for biomedical applications. As we reach the era of smart hydrogels, novel synthesis strategies are constantly sought. The advent of new technologies allowed us to prepare hydrogels with the networks formed not only by chemical or physical crosslinking alone, but through a double network, or a combination of covalent and physical entanglements. Maximizing this kind of crosslinking enables us to increase the selection of polymers wherein natural and synthetic polymers can be used as support matrices. This, in turn, results in desirable customized properties such as biocompatibility, structural stability, tunability, and stimuli-responsive swelling behavior.

While we continue to search for different approaches to hydrogel synthesis, one emerging alternative is the use of plasma-assisted processes. Plasma treatment has proven its efficiency in tuning the surface properties of biomaterials. With its rich supply of species, plasma can generate more RONS to provide more functionality to the hydrogels. Aside from that, the radicals can also be used to facilitate the network formation of the hydrogels. While the reaction mechanisms with plasma are still complex, significant progress has been made to control the plasma parameters to tune surface interactions. Hence, it is noteworthy to further investigate the influence of plasma treatment in hydrogel synthesis. As the complexity of the plasma processes and its subsequent interactions are understood, plasma-assisted procedures are poised to become a major technique in hydrogel synthesis and modification.

Finally, process design implications must be considered for hydrogel production. The process conditions must be optimized to ensure the structural integrity of the hydrogel is met. At the same time, the service life of the hydrogel must be taken into consideration to determine its degradability or long term usability. Hence, design for scalability and design for sustainability are equally important during the initial development of novel smart hydrogels.

## Figures and Tables

**Table 1:** Typical properties of biomaterials.

Property	Sample Characteristics
Biocompatibility	Non-toxic; non-carcinogenic; non-allergenic
Physical properties	Density; porosity; form; surface roughness
Chemical properties	Inert, stable, reactive, selective
Mechanical properties	Compressive; tensile; shear; impact
Scalability	Processable; sustainable; sterilizable
Service life	Stable; tunable degradation rate
Economical	Affordable; readily available

**Table 2:** Examples of chemically crosslinked hydrogels with their characteristics.

Hydrogel	Characteristics	References
AAm/NDAPM	Stimuli responsive where volume and elasticity change	[69]
HA/2-HEA/PEGDA	Porous and biocompatible; capable of sustained drug release	[70]
Acrylate-g-PHEMA	Hydrophobic with tunable hardness and swelling	[71]
AAm/MBA	Temperature-sensitive swelling with smooth surface	[72]
Xantham/Chitosan/Gelatin/PEG	High water content, porous and biodegradable wound dressing	[73]

**Table 4:** Representative works that use plasma for hydrogel synthesis and modification.

Hydrogel	Plasma Source	Operating Gas	Target Application(s)	Reference
Chitosan/acrylic acid	Plasma jet	Air	Wound healing	[153]
tPEO	Plasma jet	He	Various	[154]
Carboxymethyl cellulose/PVA	CAP	Ar, N${}_{2}$	Drug carrier	[155]
Chitosan/PVA	Glow discharge	Air, He, N${}_{2}$	Drug delivery	[156]
Chitosan/xanthan	PECVD	He, epichlorohydrin	Drug release	[157]
HEMA:DEAEMA	Plasma jet	Ar	Biosensing	[158]
Starch/Alginate	PECVD	O${}_{2}$, tetraethyl orthosilicate	Drug delivery	[159]
Gelatin	Plasma jet	-	-	[160]
Silk/fibrin	Plasma jet	Ar, N${}_{2}$	Wound healing	[161]
Gelatin/GO	DBD	Ar	Drug delivery	[162]
PVA/alginate	CAP	Ar, hexamethyldisiloxane	Drug delivery	[163]
PVA	DBD	He	-	[164]
Silicone	Glow discharge	He	Contact lens	[165]
Gelatin	Plasma jet	He	Drug delivery	[166]
NIPAAm	DBD	He	-	[167]
PVA/nanoparticle	CAP	He	Various	[168]
Chitosan/guar gum	CAP	Ar, O${}_{2}$	Drug delivery, antibiofilm	[169]
HEMA/PVP, TRIS/NVP/HEMA	PECVD	N${}_{2}$	Contact lens	[170]
PVA/Chitosan	RF discharge	Ar	-	[171]
Carboxymethyl/guar gum/PVA	Glow discharge	N${}_{2}$ and NH${}_{3}$	Drug delivery	[172]
2-hydroxyethyl methacrylate	RF discharge	C${}_{2}$H${}_{4}$, C${}_{4}$H${}_{6}$, CO${}_{2}$, NH${}_{3}$	-	[173]
Gelatin/GO	Microplasma	Ar	Cartilage reconstructive surgey	[174,175]
Fe${}_{3}$O${}_{4}$-PNIPAm	Microplasma	He	-	[176]

tPEO—poly(ethylene oxide)-based triblock copolymer; PVA—polyvinyl alcohol; HEMA—hydroxyethyl methacrylate; DEAEMA—2-(diethylamino)ethyl methacrylate; NIPAAm—N-isopropylacrylamide; PVP—poly(vinylpyrrolidone); TRIS—3-tris(trimethylsilyloxy)silylpropyl 2-methylprop-2-enoate; GO—graphene oxide; PNIPAm- poly(N-isopropylacrylamide). PECVD—plasma-enhanced chemical vapor deposition; DBD—dielectric barrier discharge; CAP—cold atmospheric plasma; RF—radio frequency.

## Data Availability

No new data were created or analyzed in this study. Data sharing is not applicable to this article.
